# Supplementary material for: Genome-wide mapping of Cas9-induced sister chromatid exchange across single and over 200 genomic sites in human cells
Source: eLife. 2026 Oct 12;15:RP112073. doi: 10.7554/eLife.112073 (PMC13657629; doi:10.7554/eLife.112073)
Supplement: Peer review history

# Editor report: eLife Assessment

**DOI:** 10.7554/eLife.112073.3.sa0

**Authors:** Wolf-Dietrich Heyer

**Affiliations:** University of California, Davis United States

**Keywords:** Compelling, Important

Using sci-L3-Strand-seq, this study shows genome-wide, single-cell evidence that DSBs triggered by CRSIPR/Cas9 are frequently resolved through sister chromatid exchange (SCE), which is undetectable with conventional whole genome sequencing approaches. This **important** work provides helpful metrics quantifying the occurrence of SCE events at targeted and repetitive genomic loci that are of particular interest to those working in gene editing, DNA repair, genome instability, or repetitive genomic loci. The analysis of reciprocal daughter-cell pairs provides **compelling** evidence for SCE events, providing evidence consistent with CDK1-TTF2-TRAIP mediated cell-cycle regulated CMG helicase disassembly and fork cleavage at unreplicated regions.

# Referee report: Reviewer #1 (Public review)

**DOI:** 10.7554/eLife.112073.3.sa1

[Editors' note: this version has been assessed by the Reviewing Editor without further input from the original reviewers. The authors have addressed the comments raised in the previous round of review.]

Summary:

This manuscript uses sci-L3-Strand-seq to map sister chromatid exchange events following CRISPR/Cas9-induced DNA damage. Because exchanges between identical sister chromatids are largely invisible to conventional sequencing, the study addresses an important blind spot in the assessment of genome editing outcomes. The authors compare single-locus Cas9 cleavage, simultaneous targeting of 237 repetitive genomic sites, and Cas9 nickase variants. They further use reciprocal daughter-cell pair analysis to ask whether Cas9-associated SCEs are copy-neutral or linked to larger structural alterations. Overall, this is a valuable study that introduces an important additional layer to the analysis of CRISPR/Cas9 repair outcomes. The central finding that Cas9-induced DSBs can trigger frequent local SCE is well supported and likely to be of broad interest.

Strengths:

The major strength of the manuscript is the application of a strand-resolved, single-cell method to a question that is difficult to address with standard genome sequencing. The evidence that a single Cas9-induced DSB can trigger strong local SCE is compelling in concept and supported by multiple guide RNAs targeting distinct loci. The reported on-target SCE frequencies, reaching up to 41%, suggest that inter-sister exchange is a substantial and underappreciated outcome of Cas9 cleavage.

Of particular interest is the comparison between single-site and multi-site targeting. The finding that 237 programmed Cas9 targets produce only mild bulk enrichment of on-target SCE but stronger enrichment in a subpopulation of cells with elevated SCE burden is interesting and may have wider biological implications, particularly if the findings extend beyond Cas9-induced SCE to spontaneous SCEs.

The reciprocal daughter-cell pair analysis is another notable feature of the study. The observation that some Cas9-associated SCEs are accompanied by structural alterations could challenge the assumption that SCE after a programmed break reflects error-free homologous recombination.

# Referee report: Reviewer #2 (Public review)

**DOI:** 10.7554/eLife.112073.3.sa2

Summary:

In this short paper, a clever single-cell Strand-seq method was used to study the number and location of sister chromatid exchange events (SCEs) in cells after CRISPR/Cas9-induced DNA double-strand breaks (DSBs). Unique as well as multiple genomic loci were targeted. Cas9-induced cuts at unique genomic locations led to statistical enrichment of SCEs at the target site, whereas Cas9 targeted at repetitive targets revealed only mild enrichment of on-target SCEs unless analysis was restricted to a subset of cells with >8 SCEs per cell. Interestingly, reciprocal daughter-cell pair analysis revealed large-scale structural alterations on some chromosomes. Whereas disruption of DNA repair genes, including LIG3, LIG4, XRCC1, and XRCC4, did not measurably alter SCE frequency per cell within 24 hrs, consistent with delayed functional loss following editing and selection against essential genes. Together, these findings demonstrate that Cas9-induced DSBs are potent local triggers of SCE at unique loci and can be associated with structural alterations, highlighting the influence of lesion type and genomic context on recombination outcomes during genome editing.

Strengths:

The data in this paper represent a very rich resource of how parental DNA template strands are distributed in paired daughter cells after various treatments. Abnormalities observed in only one of such paired daughter cells provide a novel and exciting approach to study mechanisms of DNA instability and DNA repair at a genome-wide level in general and following Cas9-induced DSB in particular.

Weaknesses:

The effect of Cas9-induced DSBs in the cells that are used will depend on the cell cycle stage of the cells that are targeted, as well as the number of times cuts are made. The latter could happen before, during, and after DNA repair reactions on one or both alleles in a diploid cell. As a result, it is very difficult to extrapolate the mechanisms of DNA instability and DNA repair from the observed genomic rearrangements. Novel approaches are needed to limit the number and timing of Cas9-induced breaks to overcome some of these limitations.

# Referee report: Reviewer #3 (Public review)

**DOI:** 10.7554/eLife.112073.3.sa3

Chovanec and Yin used their newly developed sci-L3-Strand-seq powerful method to characterize SCE after Cas9 cleavage in a human cell line, using either a single target site or an element repeated 237 times in the genome. SCE are often neglected in DNA repair analyses since they are « genetically silent ». Interestingly, the authors found enrichment of SCE at unique Cas9 sites, but only a modest enrichment of SCE when Cas9 targets 237 sites in the genome. The genetic control of SCE formation at Cas9 sites is not deliberately addressed in this paper. However, the authors found that targeted SCE seem to be enriched in a subpopulation of cells, particularly « permissive » for SCE, but the determinants of such a population are unknown. Finally, the power of the sci-L3-Strand-seq allowed the authors to characterize a specific type of SCE based on the analysis of reciprocal daughter-cell pairs' genomes that is associated with a specific type of chromosomal rearrangement compatible with the ones observed in HR defective BRCA1/2 deficient cells.

# Author comment: Author response

**DOI:** 10.7554/eLife.112073.3.sa4

**Authors:** Peter Chovanec, Shiyang He, Yi Yin

**Affiliations:** University of California, Los Angeles Los Angeles United States; University of California, Los Angeles Los Angeles United States; David Geffen School of Medicine at UCLA Los Angeles United States

The following is the authors’ response to the original reviews.

**eLife Assessment**

Using sci-L3-Strand-seq, this study shows genome-wide, single-cell evidence that DSBs triggered by CRISPR/Cas9 are frequently resolved through sister chromatid exchange (SCE), which is undetectable with conventional whole-genome sequencing approaches. This important work provides helpful metrics quantifying the occurrence of SCE events at targeted and repetitive genomic loci that are of particular interest to those working in gene editing, DNA repair, genome instability, or repetitive genomic loci. However, the evidence supporting the proposed involvement of under-replicated region/replication termination-zone resolution and TRAIP/URR-like pathways is currently incomplete and could be strengthened with an increased number of reciprocal daughter-cell pairs and by genetic or molecular perturbation, or alternatively, this can be addressed by changing the discussion.

We appreciate the editor’s and the reviewers’ recognition of Cas9-induced SCE as an important previously invisible repair outcome and of RDCP analysis as a notable feature of the study. As proposed, we incorporated the two recent Science studies and now tone down the Discussion of our RDCP observations as consistent with, rather than definitive evidence for, the TRAIP-dependent pathway. We also detail how our single-cell genomic observations complement and extend these two studies in terms of the biological significance of the CDK1-TTF2-TRAIP axis.

Specifically, these changes are in:

Discussion. We changed

“Two recent studies revealed how the CDK1-TTF2-TRAIP axis is cell-cycle regulated to trigger mitotic CMG helicase disassembly and fork cleavage: one study showed a two-fold SCE reduction in mouse ES cells (Fujisawa and Labib, 2026), while the other showed that disrupting the TRAIP-TTF2 interaction reduced common fragile site deletions (Can et al., 2026). Our observation of the "WWC-or-WCC/deletion pair" signature in wild-type cells provides, to our knowledge, the first genetic evidence of linking a deletion with SCE and revealing both W and C unreplicated template strands present in the reciprocal daughter cell, consistent with this mechanism at single-cell genomic resolution (illustrated in Fig.3), although this is limited by the observation of only one RDCP.”

While each study highlights the biological significance of the CDK1-TTF2-TRAIP axis individually for SCE and deletions, we show the coupling relationship (and further evidence of unreplicated template strands) in reciprocal daughter cells.

Fig.3. Title and legends. We changed

“Haplotype-aware analysis of observed RDCP (Pair 4, chr1) shows SV patterns at the SCE junctions consistent with the predicted RDCP signature of SCE mediated by URRs or replication termination zones (green shaded area), although the lagging strands, rather than the leading strands, must be resolved to generate these mitotic breaks.”

**Reviewer #1 (Public review):**

Summary:

This manuscript uses sci-L3-Strand-seq to map sister chromatid exchange events following CRISPR/Cas9-induced DNA damage. Because exchanges between identical sister chromatids are largely invisible to conventional sequencing, the study addresses an important blind spot in the assessment of genome editing outcomes. The authors compare single-locus Cas9 cleavage, simultaneous targeting of 237 repetitive genomic sites, and Cas9 nickase variants. They further use reciprocal daughter-cell pair analysis to ask whether Cas9-associated SCEs are copy-neutral or linked to larger structural alterations. Overall, this is a valuable study that introduces an important additional layer to the analysis of CRISPR/Cas9 repair outcomes. The central finding that Cas9-induced DSBs can trigger frequent local SCE is well supported and likely to be of broad interest. The evidence for structural complexity associated with some induced SCEs is intriguing, but the mechanistic interpretation should either be tested directly or presented more cautiously.

Strengths:

The major strength of the manuscript is the application of a strand-resolved, single-cell method to a question that is difficult to address with standard genome sequencing. The evidence that a single Cas9induced DSB can trigger strong local SCE is compelling in concept and supported by multiple guide RNAs targeting distinct loci. The reported on-target SCE frequencies, reaching up to 41%, suggest that inter-sister exchange is a substantial and underappreciated outcome of Cas9 cleavage.

Of particular interest is the comparison between single-site and multi-site targeting. The finding that 237 programmed Cas9 targets produce only mild bulk enrichment of on-target SCE but stronger enrichment in a subpopulation of cells with elevated SCE burden is interesting and may have wider biological implications, particularly if the findings extend beyond Cas9-induced SCE to spontaneous SCEs. Given that potential, the current manuscript would benefit greatly from any experiments characterizing this sub-population: are these cells in a particular cell cycle state, experiencing changes in gene expression, or do they have other unique biological properties?

The reciprocal daughter-cell pair analysis is another notable feature of the study. The observation that some Cas9-associated SCEs are accompanied by structural alterations could challenge the assumption that SCE after a programmed break reflects error-free homologous recombination.

Thank you very much for this assessment.

Weaknesses:

The number of informative RDCPs is limited, and the mechanistic interpretation of the "WWC-orWCC/deletion" signature is more suggestive than definitive. In particular, the manuscript invokes (even though only in the Discussion section) URR or replication-termination-zone resolution and discusses TRAIP-dependent CMG unloading, nuclease cleavage, and polymerase theta-mediated joining, but these pathway components are not directly tested herein. A more conservative conclusion that some Cas9-associated SCEs coincide with structural alterations is more appropriate, particularly in the Discussion and Conclusion. For example, the statement that this work provides "direct genetic evidence" for a URR-type mechanism is overstated unless supported by additional experiments or a more extensive analysis of alternative models. Similarly, while the authors explain the limitations of acute Cas9 disruption of LIG3, LIG4, XRCC1, and XRCC4, the manuscript should clarify what biological questions this experiment can and cannot answer.

Please see response to the eLife Assessment as this is a common point raised by multiple reviewers.

Additionally, we clarified what the DNA repair gene targeting experiment can and cannot answer (delayed protein loss, essential-gene selection) by adding the following text in the “Disruption of DNA repair genes at the cut site did not measurably alter SCE frequency per cell” section:

“One additional complication is that, although Cas9 RNP achieves >90% knockout efficiency in bulk assays and is therefore used as a substitute for siRNA, sorting BrdU-labeled cells in the subsequent G1 enriches for cells that escaped frameshift editing, particularly for essential genes; thus, 100% knockout in 90% of cells is not equivalent to 90% knockdown in every cell, representing a unique challenge for single-cell assays.”

**Reviewer #1 (Recommendations for the authors):**

(1) Temper the mechanistic claims about URR/TRAIP-type resolution.

The RDCP data support the conclusion that some Cas9-associated SCEs are accompanied by structural alterations and may arise through non-classical mechanisms. However, claims about TRAIPdependent CMG unloading, URR resolution, or polymerase theta-mediated joining should be framed as a model unless directly tested.

We cited mechanistic dissection of the CDK1-TTF-2-TRAIP axis, which was published since the review of the paper. While each study highlights the biological significance of the CDK1-TTF2-TRAIP axis individually for SCE and deletions, we show the coupling relationship (and further evidence of unreplicated template strands) in reciprocal daughter cells but qualified that this observation is in only one RDCP. We further tempered our claims by changing “direct evidence” to “consistent with” as we did not perturb genes involved in these processes.

(2) Clarify the impact of the small RDCP sample size.

The manuscript would be strengthened by explicitly stating how many total RDCPs were analyzed, how many SCE events were informative, and how much confidence can be placed in the estimated fraction of SCEs associated with SVs. A short table summarizing RDCP counts, SCE counts, copy-neutral events, and SV-associated events would be helpful.

A total of 15 RDCPs were recovered from close to 4,000 single cells analyzed across all conditions. We added Tab.S3 detailing SCEs in all 15 RDCPs in addition to SCE and SV breakdown in Tab.S2 (originally Tab.S1). The new Tab.S3 is cited in the “RDCP analysis reveals large-scale SVs on chromosomes with induced SCE, as well as structural alterations at Cas9-induced SCE junctions” section.

(3) Provide more detail on the "rescued" SCE calls.

Because the central conclusions rely on SCE detection, the criteria for breakpoint R-based calls versus rescued calls should be explained clearly in the main text or methods. It would be useful to know how sensitive the main conclusions are to the inclusion or exclusion of rescued calls. Is this laid out in greater detail in an additional manuscript?

We previously included an “On-target SCE identification” section in the Methods, where we described in detail the rescue of on-target SCEs missed by the initial breakpoint R calls. We also depicted calls and calls+rescues for all the conditions in Fig.S1B.

We agree with the reviewer and now expanded the description of rescued SCE calls in the main text (under the “A single Cas9 DSB induces potent local SCE” section). In brief, 50-92% of SCEs (typically >70%) across the four single-targeting sites were directly called rather than rescued, with the exception of LIG4, where only 30% were direct calls. This is because LIG4 is located only 6 Mb from the telomere and is therefore particularly prone to missed breakpointR calls in low-coverage cells. On-target rescue at individual sites is self-contained in this manuscript because our lab primarily focuses on spontaneous SCE, for which there are no expected SCE sites. However, the rescue methodology was previously implemented in the original development of sci-L3-Strand-seq to identify SCEs at centromeres.

(4) Clarify the biological interpretation of the repetitive-target enrichment.

The high-SCE subset analysis is interesting, but the manuscript should explain whether these cells have evidence of higher RNP uptake, altered cell-cycle state, greater DNA damage, or lower sequencing quality. If these possibilities cannot be distinguished, the text should state this clearly.

We agree with the reviewer. The high SCE subset does not have lower sequencing quality by coverage or background (0.3% coverage for high-SCE vs. 0.28% coverage overall, and 3% background for both high-SCE and overall). However, our current data do not allow us to distinguish among biological explanations for the elevated SCE. The original manuscript acknowledged this limitation (“Whether this reflects a cell-cycle state more permissive to both cutting and recombination, or stochastic variation in RNP uptake coupled with a recombination-prone chromatin environment, remains to be determined.”). To make this limitation more explicit and to address the possibility of sequencing quality raised by the reviewer, we have revised the text as follows: “The high-SCE subset did not show evidence of lower sequencing quality, based on either sequencing coverage (p=0.17) or background SCE levels (p=0.13). However, we cannot distinguish whether the elevated on-target SCE reflects a cell-cycle state more permissive to both cutting and recombination, or stochastic variation in RNP uptake coupled with a recombination-prone chromatin environment.”

This question may be better explored by future co-assays with sci-L3-Strand-seq; currently we cannot enrich for cells with high SCEs to characterize the molecular features of this subset of the cells using other omics approaches.

(5) Reconsider the framing of the DNA repair gene targeting experiment.

The current data do not strongly test whether LIG3, LIG4, XRCC1, or XRCC4 regulate Cas9-induced SCE, because functional protein loss is delayed and essential-gene targeting introduces selection. This section may be better framed as a negative/control observation rather than as a pathway analysis.

We agree and please refer to Public Reviews for a single-cell assay-specific explanation.

(6) Consider including some additional control experiments, for example, Cas9 without sgRNA, nontargeting sgRNA, or mock-transfected cells to make sure that some phenotypes (for example, cell-cycle arrest) directly result from DNA cleavage rather than from the transfection procedure.

We thank the reviewer for this suggestion. We have carefully considered these additional controls but have chosen not to add further experiments. Our existing Cas9 nickase experiments provide a control that directly addresses whether the observed arrest is attributable to DSB formation rather than RNP delivery/transfection. Both the D10A and H840A Cas9 nickases were delivered under the same experimental conditions as wild-type Cas9, but neither produced the cell-cycle arrest observed following DSB induction by wild-type Cas9. Thus, these experiments control for Cas9 RNP delivery while altering the nature of the DNA lesion and support the interpretation that the observed arrest is associated specifically with Cas9-induced DSBs rather than the transfection procedure itself.

(7) Figure 1: the fonts should be increased. The majority of the labels are impossible to read in a printed copy of this manuscript.

We thank the review for pointing this out. We enlarged Fig.1 fonts.

(8) Figure 1C. The pileup plots should be described and interpreted in a clear way. In its present form, it is unclear how the interpretations and conclusions are made.

We added explanation of the pileup analysis immediately following mentioning the Fig.1C pileup: “We next examined … SCEs using genome-wide pileup analysis (Fig.1C, Fig.S1B), in which we plot the total number of SCEs detected across all single cells within each 1 Mb window.”

**Reviewer #2 (Public review):**

Summary:

In this short paper, a clever single-cell Strand-seq method was used to study the number and location of sister chromatid exchange events (SCEs) in cells after CRISPR/Cas9-induced DNA double-strand breaks (DSBs). Unique as well as multiple genomic loci were targeted. Cas9-induced cuts at unique genomic locations led to statistical enrichment of SCEs at the target site, whereas Cas9 targeted at repetitive targets revealed only mild enrichment of on-target SCEs unless analysis was restricted to a subset of cells with >8 SCEs per cell. Interestingly, reciprocal daughter-cell pair analysis revealed largescale structural alterations on some chromosomes. Whereas disruption of DNA repair genes, including LIG3, LIG4, XRCC1, and XRCC4, did not measurably alter SCE frequency per cell within 24 hrs, consistent with delayed functional loss following editing and selection against essential genes. Together, these findings demonstrate that Cas9-induced DSBs are potent local triggers of SCE at unique loci and can be associated with structural alterations, highlighting the influence of lesion type and genomic context on recombination outcomes during genome editing.

Strengths:

The data in this paper represent a very rich resource of how parental DNA template strands are distributed in paired daughter cells after various treatments. Abnormalities observed in only one of such paired daughter cells provide a novel and exciting approach to study mechanisms of DNA instability and DNA repair at a genome-wide level in general and following Cas9-induced DSB in particular.

Thank you very much for this assessment.

Weaknesses:

The effect of Cas9-induced DSBs in the cells that are used will depend on the cell cycle stage of the cells that are targeted, as well as the number of times cuts are made. The latter could happen before, during, and after DNA repair reactions on one or both alleles in a diploid cell. As a result, it is very difficult to extrapolate the mechanisms of DNA instability and DNA repair from the observed genomic rearrangements. Novel approaches are needed to limit the number and timing of Cas9-induced breaks to overcome some of these limitations. The language and logic in the paper can be improved, and some of the claims seem incorrect. For example, the abstract reads "A single Cas9 cut at a unique genomic locus led to strong local enrichment of SCE at the break site, reaching up to 41% in the same cell cycle and 17% in the subsequent division, indicating that DSB repair frequently engages non-local inter-sister repair." The evidence that only a single Cas9 cut was made is lacking (see my earlier comment); it is not clear how local enrichment or non-local inter-sister repair are defined.

We agree with the limitations that Cas9-induced DSBs can be dependent on the cell cycle stage and the number of times cuts are made. We also agree that novel approaches are needed to limit the number and timing of Cas9-induced breaks to overcome these limitations, perhaps by using vfCas9 but more importantly, if new approaches to turn off Cas9 are developed. We have added a brief discussion on this limitation (in the Limitation section) and the resulting constraints on extrapolating mechanisms of DNA instability and repair from the observed genomic rearrangements. We thank the reviewer for pointing out the distinction between “a single Cas9 cut” vs. "Cas9 targeting of a single genomic locus." We went through the manuscript and revised where cutting only once was implied. We also explicitly acknowledge the possibility of multiple rounds of cutting at the same sites.

We thank the reviewer for pointing out that “non-local repair” is a non-standard term. We use it operationally to distinguish repair confined to the broken chromatid (e.g., fill-in synthesis or end joining in cis) from repair involving exchange between sister chromatids. We have added a schematic (Fig. S1A) illustrating this distinction and cited this figure immediately before where we operationally defined SCE as a “reciprocal strand switch between sister chromatids, without implying a single mechanistic pathway.” This distinction is important because, particularly for two-ended Cas9 DSBs, an SCE-like outcome could potentially arise through either HR-mediated crossover or NHEJ of DNA ends across sister chromatids; the latter may involve different genetic requirements from classical NHEJ at least in end-tethering. We have revised the manuscript to define “non-local repair” explicitly at its first use.

**Reviewer #2 (Recommendations for the authors):**

References to relevant earlier studies using Strand-seq to study SCEs are missing (PMID: 27185886 and PMID: 29348659).

We thank the reviewer for pointing this out. We added these references in the 3rd paragraph of the Introduction where we briefly review Strand-seq methods.

**Reviewer #3 (Public review):**

Summary:

Chovanec and Yin used their newly developed sci-L3-Strand-seq powerful method to characterize SCE after Cas9 cleavage in a human cell line, using either a single target site or an element repeated 237 times in the genome. SCE are often neglected in DNA repair analyses since they are « genetically silent ». Interestingly, the authors found enrichment of SCE at unique Cas9 sites, but only a modest enrichment of SCE when Cas9 targets 237 sites in the genome. The genetic control of SCE formation at Cas9 sites is not deliberately addressed in this paper. However, the authors found that targeted SCE seem to be enriched in a subpopulation of cells, particularly « permissive » for SCE, but the determinants of such a population are unknown. Finally, the power of the sci-L3-Strand-seq allowed the authors to characterize a specific type of SCE based on the analysis of reciprocal daughter-cell pairs' genomes that is associated with a specific type of chromosomal rearrangement compatible with the ones observed in HR defective BRCA1/2 deficient cells.

Strengths:

This is an interesting paper that molecularly explores sister chromatid exchanges, which represent an important challenge in molecular biology since they are genetically silent.

Thank you very much for this assessment.

Weaknesses:

A complexity of the current paper is that it heavily relies on a recently published paper (Chovanec et al 2026, NAR) describing the powerful but complex technique sci-L3-Strand-seq. Knowledge of this paper is a prerequisite to understanding the current manuscript because no reminder is provided. In addition, the current manuscript presents the use of the sci-L3-Strand-seq technique in the study of SCE after Cas9-induced DSBs, while a companion study is referred to several times for containing results about SCE in XRCC1 KO. At some point, one questions the relevance of splitting the use of sci-L3-Strandseq in different papers instead of making a single integrated one.

We appreciate this concern. The original sci-L3-Strand-seq study is an extensive methodology paper that establishes and validates various computational framework, whereas the companion study focuses on the genetic regulation of spontaneous SCE. The experimental designs and biological questions of the companion study and the present work are therefore distinct, although we draw on selected results from the companion study where they provide useful comparisons and contrasts between spontaneous and Cas9-induced SCE. The present study addresses a distinct biological question, the response to Cas9-induced DSBs, and we therefore believe that combining these studies would make the resulting manuscript unnecessarily broad and obscure their different biological questions.

We nevertheless agree that the present manuscript should be understandable without requiring detailed knowledge of either paper. We have therefore added a brief description of the sci-L3-Strandseq approach (3rd paragraph of Introduction, Fig.S1A legends, and Fig.1B legends) and clarified the relevant methodological concepts where they are first introduced. We hope to improve the self-contained nature of the manuscript so that readers need not consult the earlier NAR papers, and the companion preprint to understand the key results.

**Reviewer #3 (Recommendations for the authors):**

(1) Abstract

"Identical sisters ": redundant

"non-local" inter-sister repair: the meaning is not clear. Do the authors refer only to "inter-sister" and therefore "non-local" is redundant, or do they imply something specific by "non-local", in which case it needs to be clarified?

"237 repetitive targets": at least a slight description of this target is needed. Is it a "random" repeat, a satellite sequence, a sequence related to a transposable element ?...

We agree with the reviewer that “identical sisters” is technically redundant. However, we have retained “identical” here to emphasize the distinction between sister chromatids vs. homolog, as SCE is sometimes misconstrued as exchange between homologs and as potentially causing loss of heterozygosity. We prefer the slight redundancy here for conceptual clarity.

We thank the reviewer for pointing out that the meaning of “non-local” was unclear. As discussed in our response to Reviewer #2, we use “non-local repair” operationally to distinguish repair confined to the broken chromatid in cis from repair involving exchange between sister chromatids. We have added a schematic (Fig.S1A) illustrating this distinction and explicitly define the term at its first use in the revised manuscript. Please see our response to Reviewer #2 above for the detailed rationale.

We thank the reviewer for asking us to clarify the nature of the 237 repetitive targets. The sgRNA targets an Alu sequence and was selected from a larger screen of >20,000 sgRNAs targeting repetitive sequences occurring at >200 genomic sites. In that screen, cellular toxicity did not simply scale with the number of predicted target sites; we therefore selected this sgRNA because its intermediate phenotype allowed us to introduce a large number of programmed DSBs without either minimal perturbation or excessive loss of cells. Thus, the 237-site guide was not an arbitrarily selected Alu-targeting sgRNA. The full repetitive-element screen is beyond the scope of the present study, but we have clarified in the Abstract that these 237 sites are Alu targets and added a brief description of the guide selection in the Methods.

(2) Introduction:

"non-local outcome / non-local repair processes": The use of "non-local" is not standard and is obscure for the reader. Specify if it has any meaning or remove it.

Please see our response above to both Reviewers #2 and #3 regarding our definition and use of “nonlocal repair”.

The authors mention that replication through a DSB generates four broken ends. However, in case the DSB is reached by one replication fork before the converging one, there are only three broken ends for at least the time required for the converging fork to reach the DSB from the other side. This may influence the repair outcome.

We agree with the reviewer. If one replication fork encounters the DSB before the converging fork, a transient three-ended intermediate can exist before the second fork reaches the break. This temporal asymmetry could influence repair pathway choice, including engagement of HR, end joining, or BIR-like repair. Our assay captures the resulting SCE outcome but cannot distinguish the order in which replication forks encounter the DSB or the repair pathway engaged at these intermediate stages. We have revised the text (2nd paragraph of the Introduction) to clarify that four broken ends represent the eventual configuration after replication through the DSB, rather than necessarily a simultaneous intermediate.

(3) Results

Cell cycle arrest experiment: it seems that a control condition with no Cas9 is missing to conclude better about what looks like a G2-M arrest, but that is not clearly mentioned.

Please see our response to Reviewer #1, Recommendation 6, regarding additional controls for the cell-cycle arrest experiment. Briefly, the D10A and H840A Cas9 nickases were delivered under the same experimental conditions as wild-type Cas9 but did not produce the cell-cycle arrest observed following DSB induction, providing an internal control for RNP delivery/transfection and supporting the association of the arrest with Cas9-induced DSBs. We would also like to clarify that the observed cell-cycle arrest is primarily a G1/S, rather than G2/M, basing on the FACS signal (see revised Fig.1B legend). This is consistent with the strong G1/S checkpoint in mammalian cells and the predominantly G1 cell-cycle distribution of BJ-5ta cells.

Note that the font size in Figure 1 is too small for readability.

We have enlarged the font sizes throughout Figure 1 to improve readability.

Figure 1B, D10A and H840A conditions:

The authors mention that nicks can be converted into DSBs through the passage of the replication fork, but do not see any cell cycle defect in the conditions tested. Is it possible that the absence of effect results from the fact that the analysis is done prior to nicks being converted into DSBs? This remark notably applies to the 237 target sites experiment. It seems that controlling for cell cycle delays for longer times is needed to conclude clearly about this aspect. In case a clear absence of cell cycle delay is observed in the 237 target sites in the Cas9 nicking condition, this would suggest that replication born DSBs behave differently from "classical" two-ended DSBs and do not trigger cell cycle arrest.

We agree that the timing of nick conversion during replication could contribute to the absence of a detectable cell-cycle delay under the conditions examined. However, extending the duration of Cas9 nickase treatment or labeling would not necessarily resolve this question, because persistent Cas9 activity permits repeated rounds of nicking across successive cell cycles, making it difficult to relate a later cell-cycle phenotype to a defined replication-born lesion. More generally, we believe that the relationship between replication-associated nicks, SCE formation, and cell-cycle progression is better addressed in the context of spontaneous SCE, which is the focus of our companion study. The present study is focused on SCE following programmed Cas9-induced DSBs, and analysis of replication-born nick lesions would require precise temporal control (ideally vfCas9 nickases that can be turned off) of individual nicking events relative to replication, for which an appropriate experimental system is not currently available to us.

Figure 1C should mention somewhere the genomic location of the four targets to clearly show that they correspond to the four major SCE peaks. In addition, there is no legend for the vertical pink stripes. Finally, it might be wise to keep the same y-axis scale for better comparisons.

Figure 1 overall: it might be wise to clearly show a no Cas9 condition to clearly set the SCE baseline and show that it is independent of Cas9 induction. As of now, it is not clear whether the non-targeted SCE comes from a specific cleavage of Cas9 or not. Such an aspect could benefit from putting Figure S1C in the main Figure 1. Alternatively, results from Chovanec et al 2026 (NAR) should be better restated because the reader does not necessarily have them in mind.

We thank the reviewer for these suggestions. The expected Cas9 target positions were already indicated by vertical bars in Fig. 1C; however, we agree that this was not sufficiently clear. We have therefore revised the figure legend to explain that the vertical bars indicate the expected Cas9 target positions. The Chovanec et al. (2026, NAR) study focused entirely on spontaneous SCE, which we simultaneously map here as the background signal, rather than the on-target SCEs induced by Cas9. We hope that explicitly identifying the target locations in the revised legend makes this distinction clear and ensures that prior knowledge of the NAR study is not necessary to interpret Fig. 1C.

We have retained the individual y-axis scales because the magnitude of SCE enrichment differs substantially among conditions. Using a common y-axis scale would make several of the on-target SCE peaks difficult to visualize.

The section « Disruption of DNA repair genes at the cut site did not measurably alter SCE frequency per cell » is questionable in the results section for the following reasons:

(i) The DNA repair genes are used here as target sites for Cas9 cleavage, but are not the object of the study, but may be the object of a companion paper. This aspect is slightly misleading.

(ii) As first mentioned in this section, there is evidence strongly suggesting that inactivating DNA repair genes will not affect SCE, and this is what the authors observed.

(iii) As an alternative, one could put the emphasis on the fact that the effect of Cas9-mediated inactivation of DNA repair genes (ie LIG3) starts to be detectable only in the washout condition ie after at least one cell cycle. But in this case, this is addressing the role of DNA repair genes in Cas9-induced SCE, which is not the point of the current paper.

We thank the reviewer for this comment and agree that the original framing of this section could give the impression that these experiments were intended to test the functions of the targeted DNA repair genes in SCE. This was not our intent. Rather, these genes provided defined genomic target sites for Cas9 cleavage, and the primary purpose of the experiment was to characterize SCE associated with Cas9-induced DSBs at these loci.

As discussed in our response to the Editor Assessment, there are important limitations to using these experiments to infer the consequences of loss of the targeted proteins, including the delay between Cas9 cleavage and depletion of pre-existing protein and, and particularly in the single-cell assay, selection for cells that escape disruptive editing at essential genes. We have added text to the Results explicitly describing these limitations.

We therefore agree with the reviewer that the delayed effects observed under the washout condition should not be interpreted here as establishing a role for individual DNA repair genes in Cas9-induced SCE. We have revised the section title to “Cas9 targeting of DNA repair gene loci did not immediately alter overall SCE frequency per cell” to clarify the scope of this experiment and to avoid implying that testing the functions of the targeted DNA repair genes is a major objective of the present study.

The conditions in Table 1 need to be homogenized and better explained:

- 237 sites and 237 cuts are used: homogenize?

We thank the reviewer for spotting this. We revised both to be “237 sites”.

- May explain better the rationale for putting BrdU simultaneously with Cas9 or after 24 h and a wash.

For the single-targeting sites, we observed more SCE when BrdU was added simultaneously with the Cas9 for the same 24 hours, compared to adding BrdU in the subsequent division after a wash. Therefore, for the 237 sites, we analyzed both conditions.

- Typo in the text: 237cuts_24ws_40BrdU instead of 237cuts_24ws_BrdU

We apologize for the lack of clarity in these labels and have substantially revised the Table 1 legend. In brief, the “40” is not a typo. In the 237 sites experiments, wild-type Cas9 considerably prolonged the cell cycle. Therefore, rather than labeling with BrdU for 24 hours as in the other conditions, we extended BrdU labelling to 40 hours in the last two conditions to allow more cells to progress into the subsequent G1 for successful Strand-seq analysis. We clarified that “237 sites 24 + 16hrs BrdU” refers to the condition in which Cas9 RNP and BrdU were added simultaneously. After 24 hours of Cas9 RNP treatment, BrdU labeling was continued for an additional 16 hours (a total of 40 hours of BrdU). The “237sites 24ws40BrdU” condition is the corresponding washout condition, in which Cas9 RNP was removed after 24 hours and cells were then labeled with BrdU for 40 hours post-washout.

- 237 cuts: Are some sites more enriched in SCE than others?

Yes, some sites showed greater SCE enrichment than others. We tested whether this variation correlated with chromatin accessibility but found no significant association. This was not unexpected, as the sgRNAs predominantly target Alu elements.

- Table 1: There is a difference between 237 sites 24ws24BrdU and 237cuts24ws40BrdU, with a significant enrichment of on-target SCE for the latter condition only. Could the increase in SCE rise even more with longer BrdU exposure? In other words, does the low enrichment in SCE at target sites in the 237 sites experiment result from a non-optimal timing for the analysis?

Yes, this is possible. We did not systematically test additional treatment or labeling durations. A 24-hour Cas9 RNP treatment is typically used for Cas9 RNP-mediated knockout experiments, and we therefore initially used this duration to assess gene-editing outcomes. For Strand-seq, BrdU labeling is ideally limited to approximately one cell division. Because BJ-5ta cells have an approximately 24-hour cell cycle, extending BrdU labeling substantially beyond 40 hours could allow some cells to undergo a second round of replication and become double-labelled. We therefore did not extend BrdU labeling beyond 40 hours. Thus, the lower enrichment in the 24ws24BrdU condition may in part reflect the timing of the assay.

- Figure 2 / RDCP analysis:

Interpretation of this figure relies exclusively on the 2026 NAR paper from the authors. This, at least, should be mentioned to help the reader understand it. Once the legend restates, this figure misses clear identification of the SCE and other genomic rearrangements. For readability, maybe the full genome should be kept for the supplementary data, and only the rearranged chromosomes should be kept in the main figure so that the rearrangements are clearly visible and annotated.

We thank the reviewer for this suggestion. To make the Strand-seq plots interpretable without relying on our 2026 NAR paper, we have added an explanation of Strand-seq orientation in the third paragraph of the Introduction and in Fig.S1A. We have revised Fig.2 legends to improve readability. We have retained the whole-genome view because Strand-seq data are conventionally presented in this format and it provides important genome-wide context for interpreting the observed events. The rearranged chromosomes and events were annotated in Fig.S3.

(4) Discussion

- Most DSB never formed or did not produce SCE: how to understand this better? What would be the argument in favor of one or the other possibility?

We agree that these are two possible explanations that cannot be distinguished by the current experiment. The absence of an SCE at a targeted site could reflect either inefficient DSB formation or repair of a DSB through a pathway that does not generate an SCE. Distinguishing these possibilities would require direct measurement of cutting efficiency at individual target sites, which was beyond the scope of this study.

- The conclusion about the effect of the Cas9 nickases needs to be toned down as long as the proper timing for SCE analysis has not been performed (see comment above).

We agree and have toned down this conclusion by specifying that no significant on-target SCE enrichment was detected under the conditions tested and acknowledging that we cannot exclude SCE formation at other time points (Discuss, first paragraph).

- As much as possible, avoid the use of non-conventional acronyms like URR.

We agree and have reduced the use of non-conventional acronyms where possible. We have retained URR (under-replicated region), as the term appears seven times throughout the manuscript, but have ensured that it is clearly defined at first use.

- The discussion about the RDCP analysis in the second paragraph of the discussion should refer to Figure 3.

Thank you for pointing this out. We added this reference to Fig.3
